# Improving protein-ligand complex generation with force field guidance

**DOI:** 10.1186/s13321-026-01198-2

**Published:** 2026-05-02

**Authors:** Helen Lai, Tingyu Wang, Hassan Sirelkhatim, Joe Eaton, Howard Huang, Brad Rees, Ola Engkvist, Jon Paul Janet, Xiaoyun Wang, Alessandro Tibo

**Affiliations:** 1 Molecular AI, Discovery Sciences, R&D, AstraZeneca, Cambridge, UK; 2 NVIDIA, Santa Clara, CA US; 3 Molecular AI, Discovery Sciences, R&D, AstraZeneca, Gothenburg, Sweden; 4 Data Science and AI, Computer Science and Engineering, Chalmers, Gothenburg, Sweden

**Keywords:** Structure-based drug design, Protein–ligand generation, Diffusion models, Flow matching, Guidance, Force fields, Chemoinformatics

## Abstract

**Abstract:**

Generative models based on diffusion and flow matching have recently been applied to structure-based drug design, but their outputs often include unrealistic protein–ligand interactions that do not obey the laws of physics. We present an energy guidance framework that incorporates a molecular mechanics force field (MMFF94) directly into the sampling process. The method steers molecular generation toward more physically plausible and energetically stable conformations without retraining the underlying model. We evaluate this approach using two state-of-the-art architectures, SemlaFlow, a flow matching model and EDM, a diffusion model, on the PDBBind dataset. Across both models, energy guidance improves enthalpic interaction energy, improves strain energy by up to 75$\%$, and generates over 1000 ligands with better docking scores than native ligands. These results demonstrate that lightweight, physics-based guidance can significantly enhance generative drug design while preserving chemical validity and diversity.

**Scientific contribution:**

We introduce a novel, *training-free force field guidance* framework that steers ligand generation using empirical molecular mechanics (e.g., MMFF94) during diffusion or flow-based sampling–without modifying or retraining the base generative model (e.g., EDM or Semflaflow by [24]). Our method operates as a plug-in during inference time, leveraging energy feedback to generate poses with lower strain and having better predicted interactions with the protein structure.

Our main contributions are as follows:

- Energy-based guidance without retraining: Unlike methods that require gradients from neural affinity predictors (e.g., BADGER [26]), our approach injects classical force field feedback (MMFF94) directly during the posterior sampling step.
- Improved docking and strain metrics: In benchmarks against unconditional EDM and Semflaflow, our guided inference yields consistently better AutoDock Vina scores and lower ligand strain energy, even after optimizing the final structures using the same force field.
- Compatibility and flexibility: Because the guidance module is external, it can be applied broadly to multiple generative backbones–without retraining or architecture modifications, and can be applied to arbitrary differentiable potential energy functions.
- Theoretical guarantee of stability. We demonstrate in Appendix B that the gradient correction step corresponds to a descent step on the energy under standard smoothness assumptions. While the full sampling update also includes model-driven (and, in the diffusion case, stochastic) components, this result formalizes how the guidance term locally biases the trajectory toward lower-energy regions and provides a principled justification for its stabilizing effect.

## Introduction

Structure-based drug design (SBDD) plays a central role in modern drug discovery [3], focusing on the design and optimization of ligand molecules that exhibit strong enthalpic interaction energy to a specific protein receptor site informed by an experimentally observed or predicted 3D structure of the target. By leveraging the three-dimensional structural information of target proteins, SBDD enables the rational design of compounds that bind tightly to the target structure, for example via formation of specific interactions with amino acids in the target structure [16] (for example, hydrogen bonds) or occupying hydrophobic pockets resulting in the displacement of energetically unfavorable waters [1]. SBDD is traditionally achieved using molecular docking [17], that is using physics-inspired approaches that attempt to position a given molecule in the most favorable position relative to a static target structure and then predict the potential strength of the interaction via a combination of favorable specific interactions while (ideally) accounting for the energetic strain of the ligand molecule when adopting the proposed binding conformation. These docking programs typically incorporate force fields, parameterized potential energy surfaces for atomic systems that are used to estimate both the strength of interactions between proteins and ligands as well as the strain of the ligand.

Recent advances in “3D generation methods” leveraging diffusion or flow matching methods now allow machine learning models to create binding poses [14] or directly design potential binders conditioned on a provided target structure in a purely data-driven manner. This last category is particularly attractive as these models can potentially directly propose ligands that are complementary to the given target, eliminating the need for an additional ligand search strategies and profiling of potentially millions of ligands to find binders. However, many groups [11, 20, 29, 37] have identified that the poses generated by these methods often fail rudimentary sanity checks relating to physically achievable bond distances and angles, fail to make meaningful interactions with the targets or are simply nonphysically strained geometries. While improvements over the initial generation of such models have been made [15], to produce physically reasonable geometries it remains standard practice to minimize the proposed geometries with respect to a classical force field after generation.

## Related work

### Guided Diffusion and flow matching models

In recent years, diffusion and flow matching models have gained significant popularity and demonstrated strong generative capabilities across diverse domains such as text-to-image generation [34, 36], natural language processing [4], and molecular design for drug discovery [23, 24]. The original formulation of the diffusion and flow matching models support only unconditional generation. However, recent developments have introduced mechanisms to guide the generation process towards desired outputs. Two foundational approaches in this direction are classifier-guided diffusion and classifier-free guided diffusion.

In classifier-guided diffusion, an external classifier is trained separately to predict the target category from a given sample [45]. During inference, at each diffusion step, the gradient of the classifier’s output with respect to the current sample is computed and added to the predicted noise estimate. In contrast, classifier-free guidance does not require a separate classifier. Instead, the diffusion model is jointly trained on both conditional and unconditional data [21]. At inference time, guidance is applied by taking a weighted combination of the conditional and unconditional noise predictions. A scaling factor controls the strength of the conditioning, allowing for flexible adjustment of the generation process without relying on external models. Both methods discussed above are primarily designed for categorical conditioning, with classifier-free guidance also supporting text embeddings. However, these approaches are insufficient for our setting, where the conditioning variable is continuous in nature–for example, a molecular force field.

Another line of work extends the conditioning framework to the continuous regime by training the model to directly learn the gradient of the log-conditional density, $\nabla _{x_t}\log p(x_t \mid y)$ [6], where $x_t$ denotes the generated sample at time *t* and *y* the desiderata label. An alternative approach adopts a reinforcement learning paradigm, reformulating the iterative denoising process of a diffusion model as a multi-step Markov Decision Process (MDP). In this framework, policy gradient methods are applied to optimize the sampling trajectory such that the generated samples maximize a task-specific reward, such as human feedback [8, 47].

Although the two approaches above support continuous conditioning variables, they still require retraining the diffusion model for each new conditioning input. This limitation becomes particularly restrictive in structure-based drug design (SBDD), where different stages of a project or entirely different projects may involve varying conditioning inputs. The need to retrain the model for each new application is both time-consuming and computationally expensive.

In line with trends in molecular generation, recent work in protein conformation generation increasingly incorporates guidance and conditioning during both training and sampling. One prominent example is the work by [43], which adopts a two-stage learning approach to guide the diffusion process not only toward the data distribution but toward distributions that respect physical laws, specifically the Boltzmann distribution describing equilibrium states of physical systems. In the first stage, a baseline diffusion model is trained using classifier-free guidance, where sequence information processed via precomputed representations from ESMFold serves as the conditioning variable for the conditional score model. In the second stage, the trained diffusion model is used to compute an intermediate force, which is then used to train an intermediate force network. At inference time, this force network is applied at each diffusion step to compute force vectors that guide updates to the translational components of the protein conformation.

Similar to the approaches discussed previously, this framework requires a full retraining and reformulation of the guidance network when a different guidance objective is introduced. In agile structure-based drug design (SBDD) settings, it is often necessary to rapidly modify guidance parameters or incorporate new, domain-specific physical constraints. While the proposed framework is powerful and novel in its incorporation of molecular dynamics–based energy guidance, it may be less suitable as a plug-and-play solution for efficiently exploring diverse physical guidance objectives.

A related line of work is represented by RFdiffusion, adopts a more comprehensive conditioning framework that supports multiple types of constraints, including symmetry specification, motif scaffolding, binding target interactions, and topology-constrained design  [44]. For symmetry specification, conditioning is applied at inference time by transforming the initial random frames using symmetry operations . Symmetry is then preserved throughout the denoising trajectory by explicitly re-symmetrizing the structure at each denoising step. In contrast, the remaining conditioning modalities are incorporated during training. Motif scaffolding is implemented by masking motifs to keep them fixed during training, after which their three-dimensional coordinates are directly provided as input to guide scaffold generation at inference time. Conditioning on binding target interactions and topology-constrained design, however, requires fine-tuning the model on dedicated datasets, such as target protein–complex structures and block-adjacency representations that define the desired protein fold.

Despite its comprehensiveness and the ability to inject conditioning at inference time, the conditioning mechanisms in this framework are deeply integrated into the model’s learned representations and architecture. As a result, it remains distinct from the guidance paradigm introduced in this work, where guidance objectives are implemented as plug-and-play, modular scorers that steer the sampling process externally.

An approach that is closely related to the current guidance paradigm is ExEnDiff, which similarly focuses on augmenting the sampling process with guidance information while keeping the training workflow intact [31]. Specifically, the paper uses a set of experimental measures computed based on the conformations by leveraging a manifold constraint sampling technique. At inference time, a corrective potential term is added to the original score function, derived by numerically approximating the gradient of the log-likelihood of the measurement given the noisy sample. This enables flexible integration of various types of continuous guidance information, without the need to retrain the diffusion model. It is important to note that the current formulation is derived based on the diffusion framework and is tightly coupled with the score function. The flow matching model, however, does not explicitly learn the score function as such, but instead models the vector field that defines the probability path from a source distribution to a target distribution. Hence, the framework introduced in this paper cannot be directly applied to flow matching models as our proposed framework would.

In the following sections, we describe the molecular generation task and detail the specific form of conditioning used in this work: differentiable molecular force field descriptors. We then introduce our proposed method–an adaptation of classifier-guided diffusion–that enables flexible integration of differentiable conditioning signals during sampling, without requiring retraining of the diffusion model nor the descriptors.

### Molecular force fields

A molecular force field is a set of mathematical functions and parameters used to estimate the potential energy of a system of atoms based on their positions. Force fields are central to methods like molecular mechanics (MM) and molecular dynamics (MD) simulations. Over the years, various force fields have been developed for different applications–among the most prominent are AMBER [13], CHARMM [10], MMFF94 [19], and UFF [35]. AMBER and CHARMM are primarily tailored for large biomolecular systems such as proteins, peptides, and their interactions with ligands. While they offer high accuracy, their computational cost can be significant due to complex parameterization. This will become a major bottleneck in the current guidance framework where the force field evaluations need to be performed repeatedly during the denoising process. On the other hand, MMFF94 and UFF are designed for small, drug-like molecules and are much faster. However, UFF tends to be overly generic and is the least accurate among them, while MMFF94 shows a better balance between speed and accuracy, though it is traditionally limited to intra-ligand interactions [27]. In this work, we therefore rely on MMFF94, which we extend by conditioning it on the protein pocket. We provide a GPU implementation that enables fast and differentiable interaction modeling, making it suitable for integration into diffusion and flow matching sampling workflows.

## Methods

In this section, we present a strategy for enhancing molecular sampling from flow matching and diffusion models guided by a chemo-physics score–specifically, the MMFF94 force field [19]. Importantly, our method does not require any fine-tuning of the pretrained diffusion model; instead, we act solely at inference time. This allows for a flexible integration of domain-specific knowledge without compromising the generality of the learned generative process.

We denote with $\mathcal {X}$ the molecular space, whose elements are molecules $X\in \mathcal {X}$, each represented as a graph $X= (V, E)$, where *V* is the set of nodes (atoms) and $E \subseteq V \times V$ is the set of edges (bonds). Each node $v \in V$ corresponds to an atom and is represented as a 3-tuple:

1 $$\begin{aligned} v = (x, a, c), \end{aligned}$$

where $x \in \mathbb {R}^3$ denotes the 3D spatial coordinate of the atom, *a* is the atom type, and *c* is the formal charge. Note that both *a* and *c* are categorical variables. Each edge $e = (v_i, v_j) \in E$ corresponds to a bond between atoms $v_i$ and $v_j$ and is associated with a bond type attribute $b_{ij}$, which in our setting can be one of: single, double, triple, or aromatic.

In addition to molecules, we model proteins (pockets) using a simpler representation. Unlike molecules, proteins are often provided as PDB files where explicit bond information is typically not included and must be inferred. Therefore, denoting the protein space as $\mathcal {Y}$, each protein $Y\in \mathcal {Y}$ is represented as a set of nodes $v \in V$, following the same semantic framework as in Eq. 1. It is important to note that, throughout this paper, we do not distinguish between separate spaces for proteins and protein pockets, as the latter are regarded as a subset of the atoms comprising the original protein.

### Conditional flow matching

Conditional flow matching [2, 28, 30] is a generative framework that directly models a continuous-time transport map between the noise distribution and the data distribution via an ordinary differential equation (ODE). A conditional flow matching defines a time-dependent conditional probability distribution $p_{t \mid 1}(\cdot \mid z = (X_1, X_0))$, where $X_1\in \mathcal {X}$ and $X_0\sim p_{0\mid 1}$ are a molecule and a sample drawn from a prior distribution $p_{0\mid 1}$, respectively. A common choice for $p_{t \mid 1}$, in the case of continuous variables [28], is a Gaussian distribution centered at the linear interpolation $X_t = t X_1 + (1 - t) X_0$ with a constant standard deviation. From this conditional distribution, the conditional vector field $u(\cdot \mid t, z = (X_1, X_0))$ can be analytically derived as

2 $$\begin{aligned} u(\cdot \mid t, z ) = X_1 - X_0. \end{aligned}$$

We model the vector field in using a neural network parametrized by a set of weights $\theta$, $u_\theta : [0,1] \times \mathcal {X}\rightarrow \mathcal {X}$, and train it to reconstruct the vector field defined in Eq. 2. Instead of training the model to predict $X_1 - X_0$, we can train $u_\theta$ to reconstruct clean data $X_1$ from noisy inputs $X_t$ [12, 40], and subsequently recover the underlying vector field. For instance, in the continuous setting, the following identity holds [24]:

3 $$\begin{aligned} X_1 - X_0 = \frac{1}{1 - t} (X_1 - X_t). \end{aligned}$$

To enable the generation of molecules that bind to protein targets, we extend the vector field $u_{\theta }$ to incorporate conditioning on a protein pocket $Y\in \mathcal {Y}$. We therefore redefine the neural network as

4 $$\begin{aligned} u_\theta : [0,1] \times \mathcal {X}\times \mathcal {Y}\rightarrow \mathcal {X}, \end{aligned}$$

where $\mathcal {Y}$ denotes the space of protein pockets. To generate novel molecular samples, the vector field $u_\theta$ is integrated using a standard ODE solver. A basic Euler integration scheme is presented in Algorithm 1. As the backbone architecture, we adopt SemlaFlow [24], augmented to support conditioning on protein pockets. Details of this extension are provided in Section Protein conditioning.

**Algorithm 1:**
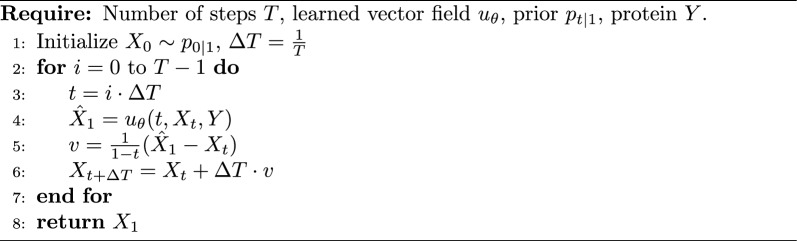
Conditional Flow Matching Sampling

### Diffusion models

Alternatively to conditional flow matching models, diffusion models [22] are another class of generative models that learn to sample complex data distributions by learning to reverse a process that adds noise to the data. For clarity and consistency throughout the paper, we slightly depart from the standard notation commonly used in diffusion models by introducing a relabeling function, $\tau (t) = \big \lfloor T\,(1 - t) \big \rceil$, where $\big \lfloor \cdot \big \rceil$ is the round to nearest integer operator, $t \in [0,1]$ denotes the normalized time and *T* is the total number of time steps. With this convention, we denote the clean sample as $X_{\tau (1)}$ and the noisy sample as $X_{\tau (0)}$. Note that this remains consistent with the typical diffusion model notation, where $X_{\tau (1)} = X_0$ (clean) and $X_{\tau (0)} = X_T$ (noisy). This choice aligns with the flow matching notation introduced in "Methods" section, enabling a unified presentation across both paradigms. Diffusion models consist of two steps: forward and reverse processes. In the forward process, a sample from the data distribution is progressively perturbed by adding noise, eventually mapping it to a simple known prior distribution $p_{\tau (0)}$ (e.g., Gaussian noise for continuous data). The reverse process is then learned via a neural network that gradually denoises the sample, reconstructing a data point from the noise.

Formally, given a data point $X_{\tau (1)}$, the forward process defines a Markov chain of *T* steps:

5 $$\begin{aligned} X_{\tau (1)} \rightarrow X_{\tau (1-\Delta T)} \rightarrow \dots \rightarrow X_{\tau (0)}, \end{aligned}$$

where $\Delta T = 1/T$ and $X_{\tau (0)} \sim p_{\tau (0)}$ for sufficiently large *T*. The reverse process is modeled by a neural network $p_\theta$, parameterized by weights $\theta$, and can be formulated in several equivalent ways: by directly estimating $X_{\tau (t + \Delta T)}$ from $X_{\tau (t)}$ [39], by predicting the noise added at each step [22], or by predicting the original clean sample $X_{\tau (1)}$ [33]. For convenience, we adopt the latter formulation, i.e., modeling

6 $$\begin{aligned} p_\theta (X_{\tau (1)} \mid X_{\tau (t)}), \end{aligned}$$

as it enables direct computation of molecular energy at each step based on the current estimate of the original structure. Additionally, we extend our model to condition on proteins, resulting in modeling

7 $$\begin{aligned} p_\theta (X_{\tau (1)} \mid X_{\tau (t)}, Y). \end{aligned}$$

As the backbone architecture, we adopt EDM [23], augmented to support conditioning on protein pockets (see "Protein conditioning" section for the details). The sampling procedure is depicted in Algorithm 2. ComputePosterior allows to samples $X_{\tau (t+\Delta T)}$ given $\hat{X}_{\tau (1)}$ and $X_{\tau (t)}$. More details about ComputePosterior can be found in Appendix A.

**Algorithm 2:**
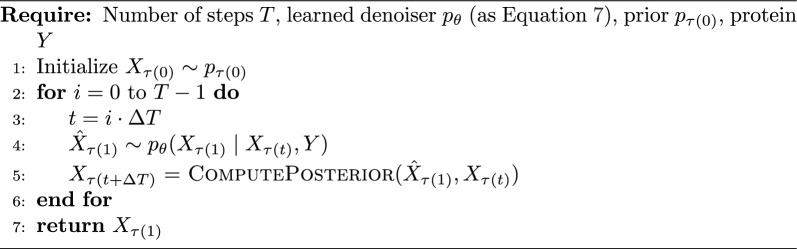
Diffusion Model Sampling

### Protein conditioning

For our diffusion and flow matching models, we adopt SemlaFlow [24] and the Equivariant Diffusion Model (EDM) [23], both extended to support protein conditioning. In this section, we describe the key architectural modifications introduced to enable conditioning on protein structures, particularly focusing on changes to the model layers.

Let us consider two tensors, $x \in \mathbb {R}^{m \times d}$ and $y \in \mathbb {R}^{n \times d}$, with dimensions $m \times d$ and $n \times d$, respectively. Here, $x$ and $y$ represent the feature vectors at any layer associated with ligand and protein atoms, respectively. The conditioning is applied within the attention layer, and its core idea can be summarized as follows:

8 $$\begin{aligned} x_i = x_i \, + \, \sum _{i\ne j} \frac{x_i - x_j}{\Vert x_i - x_j\Vert } \phi _{inv} + \, {\sum _{k} \frac{x_i - y_k}{\Vert x_i - y_k\Vert } \psi _{inv}}, \end{aligned}$$

where the right-hand summation (highlighted in blue) in Eq. 8 represents the protein conditioning we introduced. The functions, $\phi _{inv}$ and $\psi _{inv}$ are learnable mappings applied to invariant features, such as atom and bond types.

### Energy guidance

In the context of molecular generation, both diffusion models and flow matching have been leveraged to learn distributions over molecular graphs or 3D structures. However, these models are often trained solely on data likelihood objectives, potentially ignoring important physical or chemical properties that govern molecular stability. Our method addresses this gap by incorporating a chemo-physics score (MMFF94) into the inference process, biasing the generation towards physically plausible and energetically favorable molecules. Furthermore, we extended the two non-bonded interaction terms of MMFF94–van der Waals and electrostatic interactions–to account for the protein used to condition the generations. Formally, we denote the extended MMFF94 with a function mapping a protein and molecule into a real value number, $E:\mathcal {X}\times \mathcal {Y}\rightarrow \mathbb {R}$, defined as

9 $$\begin{aligned} E(X,Y) = \text {MMFF94}(X) + E_{vdW}(X,Y) +E_{Q}(X,Y), \end{aligned}$$

where the terms $E_{vdW}(X,Y)$ and $E_{Q}(X,Y)$ model the Van der Waals and electrostatic interactions between a ligand $X$ and a protein $Y$. Atom types are automatically assigned by RDKit using the MMFF94 parameterization scheme and are not inferred in a protein-specific manner. Even though MMFF94 is not a force field designed for proteins; however, we consider an extracted model system and treat it as such. We do not apply any charge modifications to the input structure. Protein charges are assigned using Schrödinger’s PrepWizard, and these charges are preserved during truncation. We do not perform any cutoffs; this is simply a reimplementation of MMFF94 in which PyTorch is used to perform gradient descent. Aside from gradient normalization, no additional form of regularization is applied. The implementation of $\text {MMFF94}(X)$ follows the original formulation [19] and reproduces the same outputs as the RDKit implementation. The implementation is available at https://github.com/MolecularAI/TorchMMFF94. *E* is then used to steer the generation of the molecules towards regions with lower energies. To this end, we modified Algorithms 1 and 2 by incorporating an additional gradient-based term, scaled by a hyperparameter $\lambda > 0$ which governs the contribution of the gradient to the overall objective (see Algorithms 3 and 4, respectively). As shown in Appendix B, when $\lambda$ is chosen below 2/*L* (with *L* denoting the local Lipschitz constant of $\nabla (E \circ f)$), the gradient-guided correction corresponds to a descent step on the energy under standard smoothness assumptions. In the full sampler, this correction is combined with model-driven updates (and stochasticity in the diffusion case), so a strict per-step decrease of the total energy is not guaranteed; however, the guidance term consistently biases updates toward lower-energy configurations.

**Algorithm 3:**
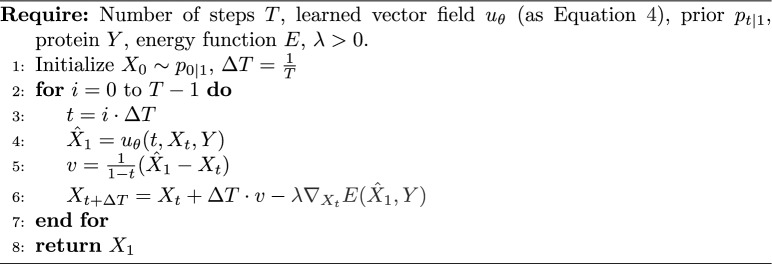
Conditional Flow Matching Sampling with Energy Guidance

**Algorithm 4:**
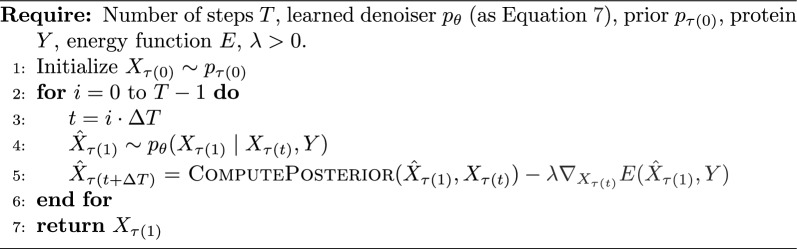
Diffusion Model Sampling with Energy Guidance

## Experiments

### Dataset

We use PDBBind [42] as our benchmark to demonstrate the quality of the generated ligands binding to proteins. PDBBind contains 19,443 protein–ligand complexes. From this dataset, we exclude 144 complexes to form our test set. These test pairs have no receptor overlap with the training set and are selected identically to those used in DiffDock [14] (i.e., timesplit_test_no_rec_overlap in the DiffDock repository). While there has been some disagreement regarding the test set chosen by the authors of DiffDock (see, e.g., [25]), our objective here is not docking but rather the generation of novel molecules within protein pockets. Therefore, this test set is suitable for our purposes.

We apply a series of preprocessing steps to the dataset using the Schrödinger suite [38]. First, we identify the protein associated with each ligand based on the distance between ligand and protein atoms. Next, we use Schrödinger’s PrepWizard to prepare both the protein and the ligand, correcting geometries and assigning appropriate protonation states. After preparation, we recompute the docking score using Glide. If the complex successfully passes through the entire pipeline and receives a negative Glide score, we retain the pair; otherwise, it is discarded. The final training set consists of 18,990 protein–ligand pairs, and the test set includes 140 pairs. Although Schrödinger Glide [18] is a commercial software package, we release the code necessary to reproduce our preprocessing pipeline. However, running it will require a valid Schrödinger license.

Following [46], for each protein we extract a binding pocket by selecting all residues that have at least one atom within 3.5Å of the native ligand and contain more than 10 atoms in total.

### Experimental setup

We evaluated our energy guidance method using two state-of-the-art generative models for molecular design: **SemlaFlow** [24], a flow matching-based generative model, and **EDM** [23], an equivariant diffusion model. Both models were initially pretrained on the **GeomDrugs** dataset [5], which contains approximately 37 million molecular conformations across more than 450,000 unique small molecules. Notably, this dataset does not include any protein structures. We adopted the default hyperparameters as reported in the respective original publications. Following pretraining, we fine-tuned each model on protein-ligand complexes from the **PDBBind** dataset, using the protein-ligand pair representation described in Section Dataset. For each protein pocket in the test set (140 targets in total), we generated 128 candidate ligands per model. To comprehensively assess the quality of the generated ligands, we employed a suite of evaluation metrics encompassing enthalpic interaction energy, chemical validity, drug-likeness, intermolecular interactions, and conformational strain:

- Glide Score: Estimated binding affinities computed using Schrödinger’s Glide, a widely used commercial docking software. Glide employs a physics-based scoring function that combines molecular mechanics with empirical terms, accounting for van der Waals interactions, electrostatics, ligand strain, hydrophobic enclosure, hydrogen bonding, desolvation effects, and other force-field-derived contributions. Its scoring function is optimized to balance computational efficiency with predictive accuracy, making it suitable for high-throughput virtual screening and lead optimization  [18].
- Vina Score: An alternative open-source binding affinities estimation method. Vina employs an empirical scoring function that estimates ligand-protein binding based on steric complementarity, hydrogen bonding, hydrophobic interactions, and torsional flexibility penalties  [41]. While its energy model is less detailed than Glide’s physics-based scoring, Vina score remains a standard benchmark in molecular docking studies.
- QED (Quantitative Estimate of Drug-likeness): A scalar score ranging from 0 to 1 that quantifies how drug-like a molecule is, with higher values indicating more favorable properties. QED integrates multiple physicochemical descriptors commonly associated with approved oral drugs, including molecular weight, lipophilicity (logP), number of hydrogen bond donors and acceptors, polar surface area, number of rotatable bonds, presence of structural alerts, and the number of aromatic rings [7].
- PoseBuster Ratio (PBR): The proportion of generated ligands that pass all PoseBuster quality checks, serving as a proxy for structural and chemical plausibility  [11].
- Better-Than-Native Count (BNC): The number of generated ligands achieving a better (i.e., lower) docking score than the corresponding native ligand.
- Validity: The percentage of generated molecules that are both syntactically correct (i.e., can be parsed into molecular graphs) and chemically interpretable. Validity is assessed using cheminformatics tools such as RDKit, which ensures that molecules can be successfully parsed from SMILES representations and can initialize a force field object (e.g., MMFF94).
- Number of Interactions: The number of hydrogen bonds formed between the generated ligands and protein, computed using the prolif library [9].
- Strain Energy: Defined as the difference in energy between the generated ligand conformation and its MMFF94-optimized geometry, normalized by the number of heavy atoms. Lower strain energy indicates more realistic and energetically favorable molecular conformations.

Importantly, we evaluate performance under two settings: (1) using the raw, unrefined ligand conformations directly output by the generative models, and (2) using conformations that have been post-processed via conditional MMFF94 minimization. In contrast to prior approaches that perform full re-docking or extensive pose refinement, our strategy is intentionally lightweight–designed to preserve the original generative intent while allowing for minor energy-based adjustments.

### Results

We report the results for SemlaFlow in Tables 1 and 2. Note that the metrics were computed directly on the generated ligands, without any re-docking. Because these scores are evaluated on the generated conformations without re-docking, they are sensitive to geometric regularity, steric clashes, and strain. As such, improvements in docking scores should be interpreted as reflecting improved physical plausibility of the generated poses, rather than a direct measure of binding affinity or docking performance under a full pose-search protocol. Table 1 reports the Vina and Glide negative ratios–that is, the proportion of generated ligands with negative scores ($\textrm{VR} < 0$ and $\textrm{GR} < 0$)–as well as the average Vina and Glide scores ($\textrm{VS}$ and $\textrm{GS}$), both expressed in kcal/mol. We also report results after applying protein-conditioned post-optimization to the generated ligands (denoted by + Opt in the Tables).

The results highlight that incorporating force-field guidance during inference leads to improved docking scores in pratice. Using MMFF94 guidance alone yields substantial improvements: $\textrm{VR}$ increases from 47.00% to 64.25%, while $\textrm{GR}$ improves even more dramatically, from 19.41% to 56.61%. Correspondingly, $\textrm{VS}$ shifts from an unfavorable 3.04 kcal/mol to a much more favorable −4.20 kcal/mol, indicating better ligand poses. Applying post-optimization alone to the baseline model without guidance also leads to significant gains, with $\textrm{VR}$ reaching 64.98% and $\textrm{VS}$ improving to −4.23 kcal/mol. However, the best performance is achieved by combining MMFF94 guidance with post-optimization, which yields the highest negative ratios for both metrics: 65.59% for $\textrm{VR}$ and 59.06% for $\textrm{GR}$. Most notably, this combined strategy produces the strongest $\textrm{VS}$ of −5.21 kcal/mol, representing an improvement of over 8 kcal/mol compared to the baseline model without guidance (3.04 kcal/mol).

**Table 1:** Vina and Glide docking score evaluation for SemlaFlow

Method	$\text {VR} < 0$	$\text {GR} < 0$	VS	GS
No guidance	47.00%	19.41%	3.04	−4.45
Guidance	64.25%	56.61%	−4.20	−4.81
No guidance + Opt	64.98%	53.85%	−4.23	−5.21
Guidance + Opt	65.59%	59.06%	−5.21	−5.03

VR < 0 denotes negative vina score ratio, GR < 0 denotes negative docking score ratio, VS denotes the average Vina score in kcal/mol, and GS denotes the average Glide score in kcal/mol

Table 2 reports complementary metrics to docking scores, namely QED, PoseBuster ratio (PBR), better-than-native ligand count (BNC), validity (Valid), number of interactions (# Interactions), and strain energy (kcal/mol). Incorporating guidance increases BNC from 296 to 696 (a 135% improvement), while substantially reducing strain energy from 6.58 kcal/mol to 1.54 kcal/mol. This reduction is particularly important, as strain energy is directly influenced by guidance during inference, in contrast to docking scores, which improve more indirectly. The post-optimization strategy without guidance also provides considerable benefits, achieving a BNC of 731 and lowering strain energy to 1.04 kcal/mol. The combination of guidance with post-optimization delivers the strongest results, with the highest BNC of 1152 (nearly a 4-fold improvement over baseline) and the lowest strain energy of 0.78 kcal/mol (an 8-fold reduction). Overall, this combined strategy reduces strain energy from 6.58 kcal/mol to 0.78 kcal/mol while maintaining robust performance across other metrics, including 34.83% PBR, 67.50% validity, and high molecular diversity (97.16%).

**Table 2:** Quality metrics for SemlaFlow

Method	QED	PBR	BNC	Valid	# Interactions	Strain Energy
No guidance	0.66	16.28%	296	69.55%	0.79	6.58
Guidance	0.66	39.33%	696	67.72%	0.77	1.54
No guidance + Opt	0.66	37.48%	731	69.55%	0.91	1.04
Guidance + Opt	0.65	34.83%	1152	67.50%	0.86	0.78

QED reports the average quantitative estimate of drug-likeness. PBR denotes the PoseBuster pass ratio. BNC gives the number of compounds whose docking scores are better than those of the native ligands. Valid indicates the proportion of valid molecules among the generated set. # Interactions specifies the average number of hydrogen bonds formed between generated ligands and their target proteins. Strain Energy reports the average conformational strain energy. The overall molecular diversity is 97.16%

Similarly to SemlaFlow, Tables 3 and 4 report the same quality metrics for EDM. Consistent with SemlaFlow, the results here demonstrate a consistent trend of improved docking scores and related metrics, with the combined guidance and post-optimization approach achieving superior outcomes in the majority of evaluation criteria. As in the SemlaFlow case, these improvements should be interpreted in light of the fact that both guidance and post-optimization reduce strain and improve geometric plausibility, which directly affects docking scores. Therefore, the observed gains reflect the effectiveness of the guidance in producing physically reasonable conformations, rather than a guaranteed improvement in intrinsic binding quality.

In Table 3, VR increases from 64.26% in the baseline to 74.45% with guidance and post-optimization, and VS improves from 1.01 kcal/mol to –4.05 kcal/mol. By contrast, GS remains relatively stable across all methods (–5.16 kcal/mol to –4.76 kcal/mol), while GR shows a marked increase from 20.84% in the baseline to 47.96% with guidance and post-optimization.

**Table 3:** Vina and Glide docking score evaluation for EDM

Method	$\text {VR} < 0$	$\text {GR} < 0$	VS	GS
No guidance	64.26%	20.84%	1.01	−5.16
Guidance	68.45%	39.49%	−2.43	−5.19
No guidance + Opt	72.81%	35.23%	−2.43	−4.92
Guidance + Opt	74.45%	47.96%	−4.05	−4.76

VR < 0 denotes negative vina score ratio, GR < 0 denotes negative docking score ratio, VS denotes the average Vina score in kcal/mol, and GS denotes the average Glide score in kcal/mol

Table 4 presents the molecule quality metrics for the EDM model. PBR increases notably from 25.23% in the baseline to 37.51% for the guidance case. BNC more than doubles from 540 to 1118, demonstrating that guidance enables the generation of significantly more molecules that outperform native binding conformations. The strain energy also shows considerable improvement, decreasing from 3.73 kcal/mol to 2.67 kcal/mol, reflecting more energetically favorable molecular conformations. When adopting post-optimization BNC increases from 540 to 801, while strain energy reduces from 3.73 kcal/mol to 1.25 kcal/mol. PBR also improves from 25.23% to 33.66%. With guidance and post-optimization, the strain energy decreases to 0.87 kcal/mol, representing a substantial 77% reduction compared to the baseline value of 3.73 kcal/mol. Note that post-optimization alone does not yield the best results in strain energy; such improvements are observed only when it is combined with guidance.

**Table 4:** Quality metrics for EDM

Method	QED	PBR	BNC	Valid	# Interactions	Strain Energy
No guidance	0.46	25.23%	540	81.26%	2.19	3.73
Guidance	0.42	37.51%	1118	77.68%	2.13	2.67
No guidance + Opt	0.46	33.66%	801	81.26%	1.83	1.25
Guidance + Opt	0.42	33.85%	1052	77.68%	1.79	0.87

QED reports the average quantitative estimate of drug-likeness. PBR denotes the PoseBuster pass ratio. BNC gives the number of compounds whose docking scores are better than those of the native ligands. Valid indicates the proportion of valid molecules among the generated set. # Interactions specifies the average number of hydrogen bonds formed between generated ligands and their target proteins. Strain Energy reports the average conformational strain energy. The overall molecular diversity is 81.30%

**Fig. 1:**
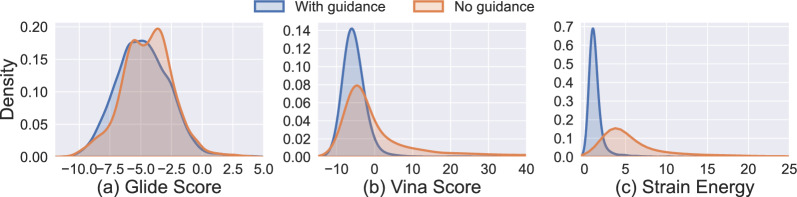
Distributions of **a** Glide Score, **b** Vina Score, and **c** Strain Energy for molecules generated with and without guidance using the SemlaFlow model. Guided molecules exhibit tighter distributions around more favorable scores and lower strain energy, whereas unguided molecules show broader distributions with higher variance and more unfavorable outliers

**Fig. 2:**
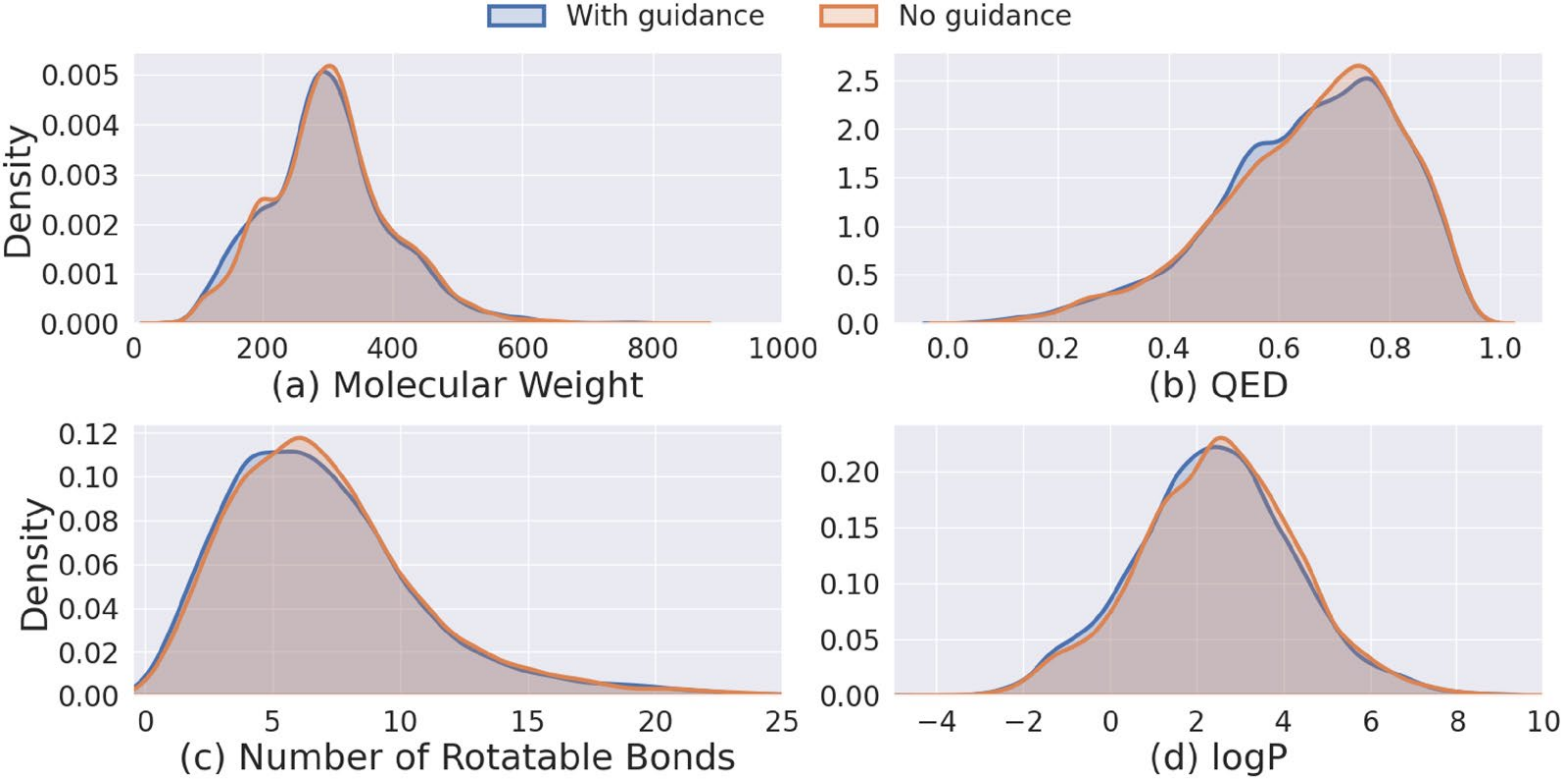
Distributions of **a** Molecular Weight, **b** QED, and **c** Number of Rotatable Bonds generated with and without guidance using the SemlaFlow model

**Fig. 3:**
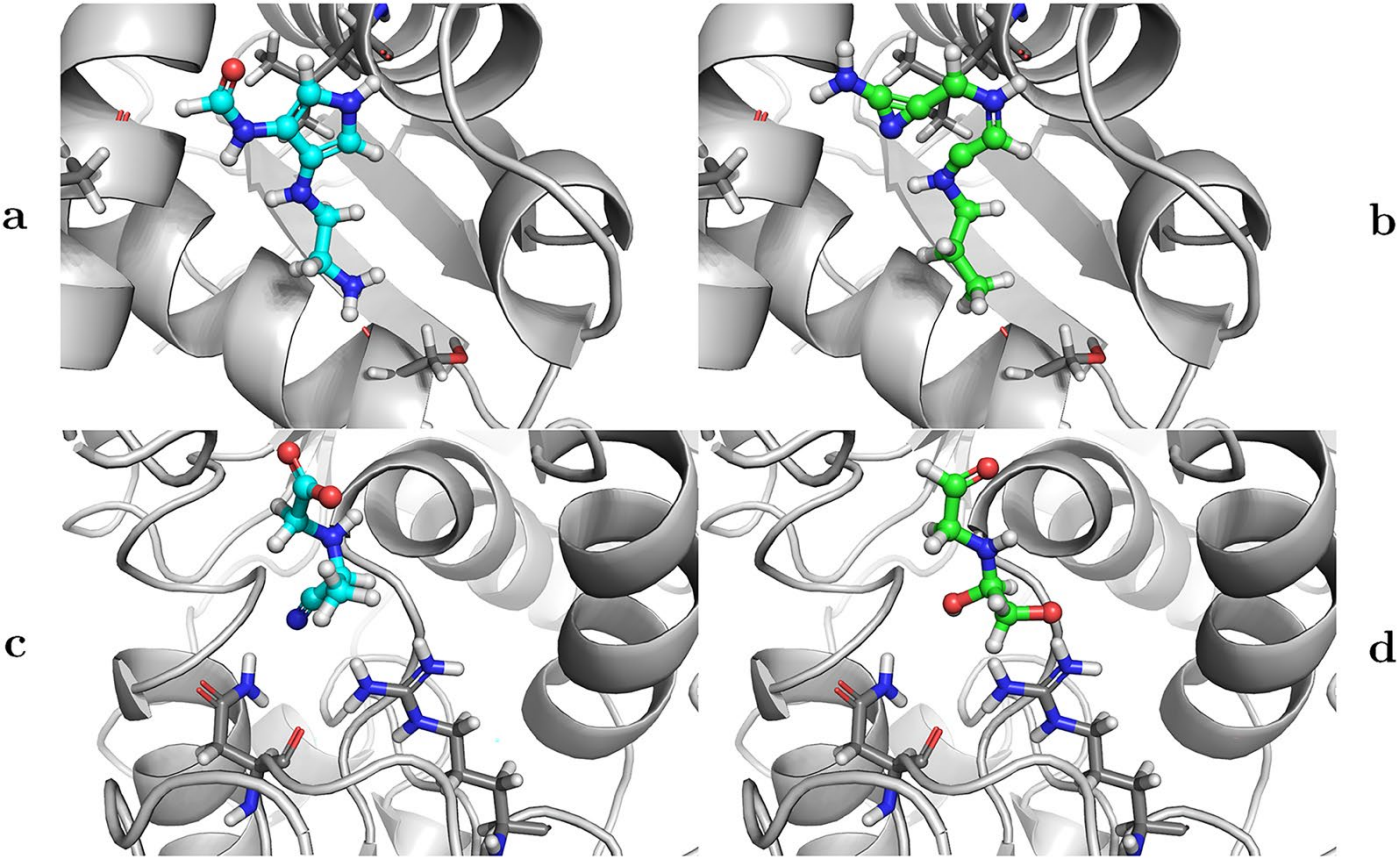
Some representative examples illustrating the impact of guidance terms on generated molecular composition from the EDM model. Results for generation with a fixed starting noise with guidance (**a** and **c**) and without guidance (**b** and **d**) for the Epstein-Barr Virus Nuclear Antigen-1 (top row, PBD:6NPM) and Aspartate semialdehyde dehydrogenase (bottom row, PBD:6C85). Proteins are shown as gray cartoons with some pocked residues shown with sticks, while generated ligands are shown with ball-and-stick representations, with guidance samples colored with cyan carbons and default samples colored with green carbons

We also plot the distributions of Glide Score, Vina Score, and strain energy for molecules generated with and without guidance using the SemlaFlow model (Fig. 1). Glide Score distributions (Fig. 2a) show that molecules generated with guidance exhibit a tighter distribution centered around lower (more favorable) scores compared to those without guidance. Vina Score distributions (Fig. 2b) follow a similar trend, where the guided molecules cluster more tightly around favorable scores (e.g., < -5 kcal/mol), whereas the no-guidance set includes a wider spread and a greater number of high (less favorable) outliers. This supports the conclusion that guidance improves the physical plausibility of the generated molecular poses, as reflected by more favorable docking scores. We emphasize that these improvements primarily capture geometric and energetic regularization effects, which are directly reflected in the docking scoring functions. Strain Energy distributions (Fig. 2c) reveal that the guided group tends to produce molecules with significantly lower strain energy. The distribution is sharply peaked around 2–3 kcal/mol for guided molecules, while the unguided set shows a broader distribution with a heavier tail, suggesting a higher incidence of conformational strain in the absence of guidance. In addition, as shown in Fig. 2, to better understand the impact of the proposed guidance framework on 2D molecular metrics, we examine the distributions of several chemical properties, including molecular weight, QED, number of rotatable bonds, and logP, for molecules generated by the SemlaFlow model. A key distinction between these properties and the previously discussed metrics is that the former depend solely on the 2D molecular graph, whereas the latter also depend on the 3D coordinates, which are directly optimized by the guided sampling algorithm. We observe no significant shift in the distributions of these 2D chemical properties between guided and non-guided molecules. This result is expected, although the current algorithm does not explicitly constrain modifications to the 2D graph, such changes occur relatively infrequently. Moreover, as illustrated in Fig. 2, when changes to the 2D graph do occur, the guidance does not induce substantial alterations in these properties. This behavior is consistent with the design of the method, as the guidance is applied directly to the 3D coordinates, and any changes to the 2D graph arise indirectly through the coupling between 3D and 2D information within the flow-matching model. Finally, Fig. 3 depicts some examples showing the impact of the energy guidance.

## Conclusion

This study introduces a novel energy-guided framework for protein-conditioned molecular generation that integrates physics-based MMFF94 force field constraints into diffusion and flow matching models. Our approach extends the MMFF94 force field to explicitly model protein-ligand interactions through van der Waals and electrostatic terms, enabling gradient-based steering during generation. It offers a lightweight yet effective alternative to existing methods that depend on computationally intensive re-docking or pose refinement. A comprehensive evaluation on the PDBBind dataset demonstrates substantial improvements across critical drug discovery metrics. Most notably, strain energy was reduced by 88% for SemlaFlow (6.58 to 0.78 kcal/mol/heavy atom) and 77% for EDM (3.73 to 0.87 kcal/mol/heavy atom), indicating significantly more energetically favorable conformations. Better-than-native counts increased by factors of 3.9$\times$ and 1.9$\times$, respectively, while enthalpic interaction energy showed dramatic improvements, with negative Vina score ratios increasing from 47.00% to 65.59% for SemlaFlow. Compared to baseline models, where the loss function only encourages the model to match the data distribution in the training set, force-field guidance pushes samples toward physically plausible regions, leading to better docking scores out-of-the-box. The consistency of improvements across two distinct generative architectures establishes the broad applicability of our energy guidance approach. In addition to empirical improvements, our theoretical analysis (Appendix B) proves that the gradient-based guidance term admits a standard descent guarantee for $0< \lambda < 2/L$, providing a theoretical justification for its stabilizing effect within the overall sampling procedure. Importantly, these quality enhancements are achieved while maintaining high molecular diversity (>80%) and chemical validity, ensuring that improved binding characteristics do not restrict chemical space exploration.

## Editorial Policies for:

Springer journals and proceedings: https://www.springer.com/gp/editorial-policies

Nature Portfolio journals: https://www.nature.com/nature-research/editorial-policies

*Scientific Reports*: https://www.nature.com/srep/journal-policies/editorial-policies

BMC journals: https://www.biomedcentral.com/getpublished/editorial-policies

## Figures and Tables

**Fig. 4:**
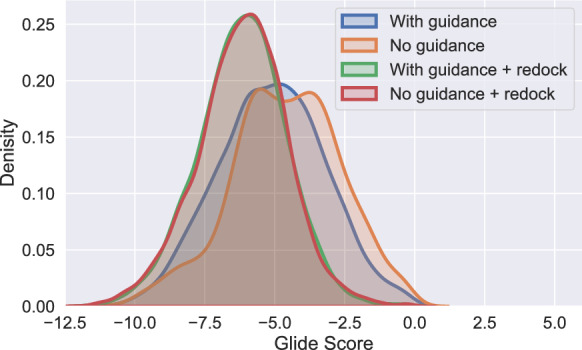
Distribution of Glide scores for generated compounds from models with and without guidance, restricted to cases where the 2D molecular graph differs between the two models. We additionally report the Glide score distributions for the redocked generated compounds in both settings

**Fig. 5:**
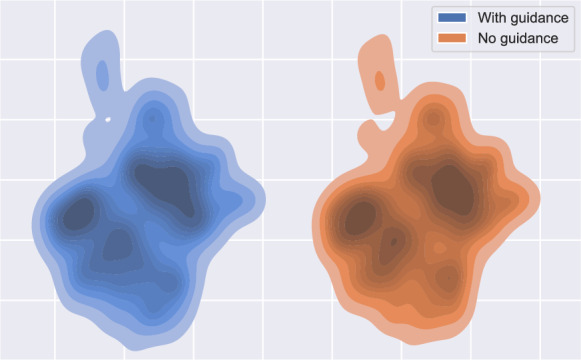
UMAP projection of ECFP4 fingerprints for molecules generated by the SemlaFlow model with guidance and without guidance. For visualization purposes, the guided distribution is artificially shifted to the left along the x-axis. The strong overlap between the two distributions indicates that both models explore a similar chemical space

**Fig. 6:**
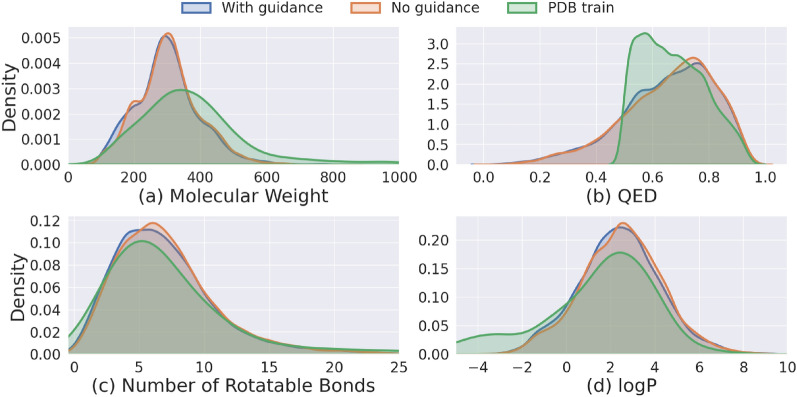
Distributions of chemical properties for guided, non-guided, and training molecules

**Fig. 7:**
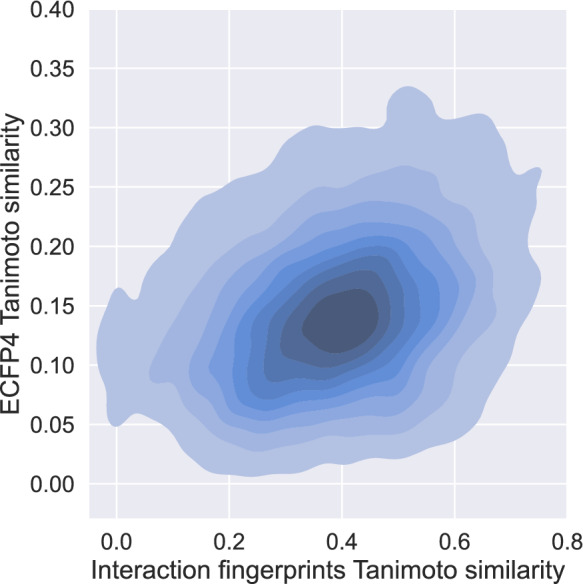
Two-dimensional distribution of Tanimoto similarities between native and generated ligands. The x-axis shows the similarity computed from protein–ligand interaction fingerprints, while the y-axis shows the similarity computed from ECFP4 fingerprints (1024 bits). Although generated ligands are often structurally dissimilar to native ligands, many retain similar interaction patterns with the protein

## Data Availability

Data and code are publicly available at the GitHub repository https://github.com/wangxiaoyunNV/NV-AZ-DrugDiscovery.

## Diffusion models

In this section, we provide the details of the ComputePosterior function from Algorithm 2, presented in "Diffusion models" section. The ComputePosterior function models the distribution $\mathcal {N}(X_{\tau (t+\Delta T)}; \mu _t(\hat{X}_{\tau (1)}, X_{\tau (t)}), \tilde{\beta }I)$, where

$$\mu _t(\hat{X_1}, X_{\tau (t)}) = \frac{\sqrt{\bar{\alpha }_{\tau (t+\Delta T)}} \, \beta _{\tau (t)}}{1 - \bar{\alpha }_{\tau (t)}} \, \hat{X}_{\tau (1)} + \frac{\sqrt{\alpha _{\tau (t)}} \, (1 - \bar{\alpha }_{\tau (t+\Delta T)})}{1 - \bar{\alpha }_{\tau (t)}} \, X_{\tau (t)},$$

and

$$\tilde{\beta } = \frac{1 - \bar{\alpha }_{\tau (t+\Delta T)}}{1 - \bar{\alpha }_{\tau (t)}} \, \beta _{\tau (t)}, \quad \alpha _{\tau (t)} = 1 - \beta _{\tau (t)}, \quad \bar{\alpha }_{\tau (t)} = \prod _{s={\tau (1-\Delta T)}}^{\tau (t)} \alpha _s.$$

The values $\beta _{\tau (t)}$ are typically chosen deterministically, with a common choice being the cosine schedule [33].

## Theoretical insights

### Lemma 1

(Descent lemma) Let $g:\mathbb {R}^n\rightarrow \mathbb {R}$ be continuously differentiable with *L*–Lipschitz gradient:

$$\Vert \nabla g(u)-\nabla g(v)\Vert \le L\Vert u-v\Vert \quad \forall \,u,v\in \mathbb {R}^n.$$

Then for all $x,y\in \mathbb {R}^n$,

$$g(y)\;\le \; g(x)\;+\;\nabla g(x)^\top (y-x)\;+\;\tfrac{L}{2}\,\Vert y-x\Vert ^2.$$

### Proof

Fix $x,y\in \mathbb {R}^n$ and define the line segment $\gamma (t)=x+t(y-x)$ for $t\in [0,1]$. By the Fundamental Theorem of Calculus applied to the scalar function $t\mapsto g(\gamma (t))$,

$$g(y)-g(x)\;=\;\int _0^1 \frac{d}{dt}\,g(\gamma (t))\,dt \;=\;\int _0^1 \nabla g(\gamma (t))^\top \gamma '(t)\,dt \;=\;\int _0^1 \nabla g(\gamma (t))^\top (y-x)\,dt.$$

Add and subtract $\nabla g(x)$ inside the integrand:

$$g(y)-g(x) =\nabla g(x)^\top (y-x) +\int _0^1 \bigl (\nabla g(\gamma (t))-\nabla g(x)\bigr )^\top (y-x)\,dt.$$

By Cauchy–Schwarz and the Lipschitz property of $\nabla g$,

$$\bigl |(\nabla g(\gamma (t))-\nabla g(x))^\top (y-x)\bigr | \;\le \;\Vert \nabla g(\gamma (t))-\nabla g(x)\Vert \,\Vert y-x\Vert \;\le \; L\,\Vert \gamma (t)-x\Vert \,\Vert y-x\Vert \;=\; L\,t\,\Vert y-x\Vert ^2.$$

Therefore,

$$g(y)-g(x) \;\le \;\nabla g(x)^\top (y-x)\;+\;\int _0^1\,L\,t\,\Vert y-x\Vert ^2\,dt \;=\;\nabla g(x)^\top (y-x)\;+\;\tfrac{L}{2}\,\Vert y-x\Vert ^2,$$

which is the claimed inequality. $\square$

### Theorem 1

Let $E: \mathbb {R}^m \rightarrow \mathbb {R}$ and $f: \mathbb {R}^n \rightarrow \mathbb {R}^m$ be $C^1$ functions, and define the composite

$$g(x) := E(f(x)), \quad x \in \mathbb {R}^n.$$

Suppose *g* is *L*–smooth, i.e.,

$$\exists L > 0 \;\; \text {such that} \;\; \Vert \nabla g(x) - \nabla g(y)\Vert \le L\Vert x-y\Vert , \quad \forall x,y \in \mathbb {R}^n.$$

Then it follows that

B1 $$\begin{aligned} E(f(x - \lambda \nabla g(x))) \le E(f(x)), \quad 0< \lambda < \tfrac{2}{L}, \end{aligned}$$

with strict inequality whenever $\nabla g(x) \ne 0$.

**Step 1 (Descent lemma).** For any *L*–smooth function *g*, we have

B2 $$\begin{aligned} g(y) \le g(x) + \nabla g(x)^\top (y-x) + \frac{L}{2}\Vert y-x\Vert ^2. \end{aligned}$$

—

**Step 2 (Gradient step).** Choose

$$y = x - \lambda \nabla g(x), \quad \lambda > 0.$$

Substituting into (B2) gives

$$\begin{aligned} g(x - \lambda \nabla g(x))&\le g(x) + \nabla g(x)^\top (-\lambda \nabla g(x)) + \frac{L}{2}\Vert \!-\lambda \nabla g(x)\Vert ^2 \\&= g(x) - \lambda \Vert \nabla g(x)\Vert ^2 + \frac{L\lambda ^2}{2}\Vert \nabla g(x)\Vert ^2. \end{aligned}$$

—

**Step 3 (Condition on step size).** Rearranging yields

B3 $$\begin{aligned} g(x - \lambda \nabla g(x)) \le g(x) - \Bigl (\lambda - \tfrac{L\lambda ^2}{2}\Bigr )\Vert \nabla g(x)\Vert ^2. \end{aligned}$$

If $0< \lambda < \tfrac{2}{L}$, then the coefficient $\lambda - \tfrac{L\lambda ^2}{2}$ is strictly positive. Hence: - If $\nabla g(x) \ne 0$, inequality (B3) is strict:

$$g(x - \lambda \nabla g(x)) < g(x).$$

- If $\nabla g(x) = 0$, then *x* is a stationary point and equality holds:

$$g(x - \lambda \nabla g(x)) = g(x).$$

—

**Conclusion.** Since $g(x) = E(f(x))$, we deduce that under the *L*–smoothness assumption on *g*, one gradient step yields

$$E(f(x - \lambda \nabla g(x))) \le E(f(x)), \quad 0< \lambda < \tfrac{2}{L},$$

with strict inequality whenever $\nabla g(x) \ne 0$.

## Additional experiment on redocking

Figure 4 shows the distribution of Glide scores for generated compounds from SemlaFlow models trained with and without guidance without post-optimization, restricted to cases where the 2D molecular graph differs between the two models. In addition, Glide score distributions are reported for the redocked generated compounds in both settings. While redocked poses achieve better scores on average, the guided model consistently produces higher-quality poses than the unguided model across both original and redocked evaluations. This observation also indicates that there remains room for further improvement.

## Generated chemical space

We analyzed the chemical space explored by the SemlaFlow model by projecting the ECFP4 fingerprints of molecules generated with and without guidance into a two-dimensional space using UMAP [32] (see Fig. 5). For visualization clarity, the distribution corresponding to the guided case was artificially shifted to the left along one the x-axis. The resulting projections show no significant differences between the two distributions, indicating that both guided and unguided models explore a similar chemical space. This behavior is expected, as the steering happens only at inference time on a model trained on one dataset. Consequently, the improvements observed when guidance is applied do not arise from changes in chemical space coverage, but rather from differences in the generated molecular geometries, as reflected, for example, in improved Glide scores.

## Comparison of 2D chemical properties between training and generated molecules

As shown in Fig. 6, the distributions of molecular weight, number of rotatable bonds, and logP for the generated molecules closely resemble those of the training set. The primary difference is that the training distribution exhibits a broader spread, with outliers corresponding to higher molecular weight, a larger number of rotatable bonds, and lower logP values. Nevertheless, the majority of the probability mass in all cases remains centered around similar ranges. For the QED score, a cutoff of 0.5 was applied during preprocessing of the training data, resulting in a sharp termination of the corresponding distribution at this value. In contrast, the distributions of QED scores for the generated molecules, both with and without guidance, are noticeably smoother. Despite this preprocessing-induced difference, the majority of molecules across all three datasets exhibit QED scores above 0.5. Taken together with the other three metrics, these results indicate that the model, both with and without guidance, effectively captures the chemical space and the drug-likeness characteristics represented in the training set.

## Interaction fingerprint similarity

Our generative model is not constrained to reproduce the same binding motifs or to generate ligands that are structurally similar to native compounds. To quantify the relationship between generated and native ligands, we computed the Tanimoto similarity for ligands bound to the same protein structures using two complementary descriptors: ECFP4 fingerprints with 1024 bits and protein–ligand interaction fingerprints [9], which explicitly encode ligand–protein interactions. In both cases, values close to one indicate high similarity. Figure 7 shows the two-dimensional distribution of these similarities, with interaction fingerprint similarity on the x-axis and ECFP4 similarity on the y-axis. While the structural similarity between generated and native ligands is generally low, many generated compounds preserve interaction patterns that are similar to those of the native ligands, indicating that the model can recover relevant binding interactions despite producing chemically diverse structures.

## Declarations

### Use of LLMs

LLMs were exclusively used for text cleanup purposes.
